# Exploring anti-corruption, transparency, and accountability in the World Health Organization, the United Nations Development Programme, the World Bank Group, and the Global Fund to Fight AIDS, Tuberculosis and Malaria

**DOI:** 10.1186/s12992-020-00629-5

**Published:** 2020-10-20

**Authors:** Jillian Clare Kohler, Andrea Bowra

**Affiliations:** 1 Leslie Dan Faculty of Pharmacy, University of Toronto, 144 College Street, Toronto, ON M4R 1V5 Canada; 2 Munk School of Global Affairs, University of Toronto, 1 Devonshire Place, Toronto, ON M5S 3K7 Canada; 3 WHO Collaborating Centre for Governance, Accountability, and Transparency in the Pharmaceutical Sector, University of Toronto, 144 College Street, Toronto, ON M4R 1V5 Canada; 4 Dalla Lana School of Public Health, University of Toronto, 155 College Street, Toronto, ON M5T 3M7 Canada

**Keywords:** Anti-corruption, International organizations, Good governance, Transparency, Accountability

## Abstract

Corruption is recognized by the global community as a threat to development generally and to achieving health goals, such as the United Nations Sustainable Development Goal # 3: ensuring healthy lives and promoting well-being for all. As such, international organizations such as the World Health Organizations and the United Nations Development Program are creating an evidence base on how best to address corruption in health systems. At present, the risk of corruption is even more apparent, given the need for quick and nimble responses to the COVID-19 pandemic, which may include a relaxation of standards and the rapid mobilization of large funds. As international organizations and governments attempt to respond to the ever-changing demands of this pandemic, there is a need to acknowledge and address the increased opportunity for corruption.

In order to explore how such risks of corruption are addressed in international organizations, this paper focuses on the question: How are international organizations implementing measures to promote accountability and transparency, and anti-corruption, in their own operations? The following international organizations were selected as the focus of this paper given their current involvement in anti-corruption, transparency, and accountability in the health sector: the World Health Organization, the United Nations Development Program, the World Bank Group, and the Global Fund to Fight Aids, Tuberculosis and Malaria. Our findings demonstrate that there has been a clear increase in the volume and scope of anti-corruption, accountability, and transparency measures implemented by these international organizations in recent years. However, the efficacy of these measures remains unclear. Further research is needed to determine how these measures are achieving their transparency, accountability, and anti-corruption goals.

## Introduction

The United Nations Office of the High Commissioner on Human Rights has underscored that corruption is an “enormous obstacle to the realization of all human rights,” and has stated that transparency, accountability, non-discrimination and meaningful participation are effective means to fight corruption [1]. Corruption, defined by Transparency International is, “the misuse of entrusted power for private gain” [2], fuels inequity as it skews how resources are allocated and distributed. Its cost is massive: every year, US$2.6 trillion are stolen - more than 5% of the global gross domestic product (GDP), with an additional US$ 1 trillion paid in bribes [3]. A 2019 report by the International Monetary Fund (IMF) further found that if all countries were to reduce levels of corruption, $1 trillion in lost tax revenues could be gained constituting 1.25% of the global GDP [4].

Corruption can be understood as a global wicked problem, which is characteristically described as being challenging and “influenced by a constellation of complex social and political factors” [5]. Corruption manifests in many forms; it may, for instance, be petty or grand, and it transcends all jurisdictional borders. Although there is seldom a single identifiable cause of corruption, certain factors may contribute to its manifestation including poverty, low social and economic status of public officials, and insufficient or an absence of institutional transparency and accountability mechanisms.

Corruption has, in light of this, been gaining traction as a policy issue for many international organizations. During the past two decades, there has been a veritable shift in the number of international organizations (IOs) addressing corruption. This shift can be attributed to a number of factors including the rising monetary costs of corruption as mentioned above, increased media attention on corruption, and an increase in public perceptions of corruption as ‘detrimental’ to society [6, 7]. The topic of corruption and its impact on development goals is no longer relegated to quiet conversations amongst policy makers and government officials. It is not being pushed out of public discourse with the view that it is simply, “*too complex*” or “*too political*.” Instead, IOs have developed anti-corruption policies and practices as well as corresponding institutions to oversee compliance with anti-corruption efforts.

### Corruption and the health system

IOs such as the World Health Organization (WHO), the United Nations Development Programme (UNDP), the World Bank Group (World Bank), and the Global Fund to Fight Aids, Tuberculosis and Malaria (Global Fund) are building an evidence base on what anti-corruption, transparency and accountability (ACTA) mechanisms can best reduce corruption in the health system. In doing so, they are aiming to contribute to programing that is in line with the global Sustainable Development Goals (SDGs), such as SDG# 3 “Good Health and Well-Being” and SDG #16 “Peace, Justice and Strong Institutions,” which includes the sub-target 16.5 “(*to) substantially reduce corruption and bribery in all their forms*. In 2019, these IOs initiated a global effort with broad stakeholder representation to construct a proposed ACTA Alliance for Health that seeks, amongst other objectives, to build up human capacity in anti-corruption and health and to monitor and evaluate ACTA measures in health [8, 9].

The justification for this focus on corruption in health is compelling, as there is ample evidence about how the health sector provides an optimal breeding ground for corruption to thrive [10, 11]. The embezzlement of public health budgets and kickbacks in the pharmaceutical procurement process, as but two examples, can result in wastage that limits access of a population to essential health services and medicines [10, 12–14].

The current COVID-19 pandemic has amplified the risks that corruption poses to the health sector. Health systems around the world are facing unprecedented and unpredictable demands on potentially already fragile systems [15]. The rapid responses from IOs and governments as they attempt to reduce the spread of the disease and attend to those who are already suffering, though necessary, are creating conditions that may be highly conducive to corruption [15]. Such conditions include flexibility in responses, the simplification of procurement controls, the rapid flow of large funds, and the need for urgent responses [15–17]. As a result, corruption and fraud are already revealing themselves in the forms of price gouging and flawed and falsified goods [16, 18–20]. This threatens to undermine the sustainability of health systems and have negative impacts that could persist long after the outbreak itself is controlled [17].

But while a focus on ACTA in health warrants examination, specifically during a global pandemic, before advancing this agenda there is value in stepping back to understand first what institutions and mechanisms IOs have in place to advance ACTA in their operations more generally. What makes this messy at times is that anti-corruption efforts have often been taken up by the global community within the framework of good governance. Given this, a closer examination of what good governance means conceptually and how it is institutionally implemented is of value for more informed policy generation in ACTA generally and in relation to the health sector, in particular. Accordingly, this paper focuses on the question: How are IOs implementing measures to promote accountability and transparency, as well as anti-corruption, in their own operations?

### Paper organization

This paper is organized in the following way: First, the topic is introduced broadly, and a brief discussion about how corruption can impact health goals follows, as above. Next, the methods are described. Then the importance of good governance as a mechanism to reduce corruption is discussed. Next, a description of examples of ACTA mechanisms within the WHO, UNDP and the Global Fund, and the World Bank are described. Lastly, findings and conclusions are provided.

## Methods

This research consisted of a targeted website review as well as an academic literature review in order to explore and synthesize the ACTA measures and processes employed by the following four IOs: the Global Fund, the WHO, the UNDP, and the World Bank. First, we conducted a targeted website review of the Global Fund, WHO, UNDP, and World Bank websites. This review was conducted between September 2019 – October 2019, and again in June 2020. Materials extracted included organizational reports, policies, internal and external audits, as well as other relevant documents. The website review was then supplemented by a rapid literature review searching the following databases: SCOPUS, Medline, Google, and Google scholar. Search strategies were utilized with a combination of the following keywords: (“UNDP” OR “WHO” OR “World Bank” OR “Global Fund”) AND (“corruption” OR “anti-corruption” OR “transparency” OR “accountability” OR “good governance”). No restrictions were applied based on geographical location, but searches were limited to articles published between 2000 and 2020 to ensure the most relevant and current material was captured.

Titles and abstracts were then examined to ensure the following criteria were met: 1) the article addressed one of the four specified institutions; 2) the article addressed the measures implemented by the organization; and 3) the article was written or translated into English. All documents were then analyzed, and the ACTA measures employed by the IOs were extracted and summarized to be presented as below.

## The importance of good governance as a mechanism to reduce corruption

Prior to examining the ACTA measures implemented by IOs, we first discuss the conceptual framework of good governance, particularly, accountability and transparency. These concepts have been applied programmatically in countries by the WHO, particularly in terms of its Good Governance for Medicines Initiative, the Democratic Governance Program of the UNDP and others, since they are often more palatable than anti-corruption to government officials, as public policy areas of focus [21]. Indeed, governing institutions are better able to respond to community needs and demands when their processes are inclusive, transparent, accountable to all stakeholders, and responsive to the demands of the governed [22]. The thinking here is that accountability and transparency can more likely illuminate areas where corruption may be present, there may be corruption risks, and/or inefficiencies [23].

In an evidence review of causes of corruption, the Organization for Economic Co-operation and Development (OECD) found that weak governance is “… one of the fundamental leading causes of corruption” [24]. Good governance is accordingly viewed as a credible conceptual framework to operationalize anti-corruption efforts and can contribute to achieving more sustainable social and economic development [25]. Handl underscores that “sustainable development is achievable only in a social setting that allows for public access to information … and governmental accountability [26].” What is more, lacking good governance in international organizations has been linked, rightly or wrongly, to corruption and the inefficiency of international financial institutions [27, 28]. As Thomas describes, “the IFIs [international financial institutions] have defined good governance as the elimination of corruption through the establishment of the rule of law and the efficiency and accountability of the public sector” [28].

The focus on good governance is therefore of value if we consider it as a *sine qua non* for advancing efficient and effective healthcare systems and for mitigating mismanagement [29]. It is also regarded as one of the key building blocks of the health system, helping to ensure that adequate policies, effective oversight, and strong accountability mechanisms are in place for its proper design and management. The complexities of the health system thus require tailored governance approaches to identify vulnerabilities to corruption, waste, mismanagement, and fraud. Anticorruption efforts that translate good governance principles have been considered as a “gold standard” for tackling sector vulnerabilities. Savedoff and Hussmann have found that, “corruption in healthcare is less likely in societies where there is broad adherence to the rule of law, transparency and trust, and where the public sector is ruled by effective civil service codes and strong accountability mechanisms” [30]. Good governance, such as accountability and transparency, allows citizens to hold authorities accountable for better development results, since it encourages civic engagement in the policy-making process and increases transparency [31]. It guides the creation and implementation of anticorruption strategies and tactics.

Yet as Vian and Kohler emphasize, how stakeholders define and implement transparency and accountability efforts can be vastly different across countries and institutional contexts in the health sector [32]. Even so, they propose definitions from existing literature. They explain that accountability can be understood as those mechanisms that make institutions responsive to their particular publics. It requires institutions or organizations to be accountable to those who will be impacted by their decisions [32]. Accountability can reduce corruption and other abuses, assure compliance with standards and procedures, and improve performance and organizational learning [33]. In addition, accountability requires institutions to justify their results to internal and external monitors and stakeholders and impose sanctions for nonperformance or corrupt behavior.

Vian and Kohler also illuminate that accountability and transparency are essentially coupled. Accountability requires transparency and vice versa. Here, transparency is understood as when citizens are informed about how and why public policy decisions are taken. To understand how public decisions are made requires information about the procedures followed and the criteria used by policymakers to reach decisions. Understanding why decisions are made requires disclosure of the information drawn on by policy makers and revelation of the arguments adduced in favour and against particular decisions [34]. Government transparency can further be understood as the level of access to government information which is made available to the population.

Still, the use of good governance as an entry point to address corruption is not a straight-forward enterprise. As noted earlier, anti-corruption efforts and good governance often become entangled together. Furthermore, the concept of good governance holds many meanings. Often these meanings are based on the mechanisms through which it can be achieved, such as participatory democracy, transparency, accountable, efficient public services, and the presence and enforcement of civil rights [35]. Other definitions are based more on specific outcomes, such as assuring that the most marginalized groups in society have a voice and receive fair, equitable treatment.

## ACTA institutions/mechanisms within IOs

In order to understand how corruption is being addressed by IOs, we set forth to find examples of how the WHO, the UNDP, the Global Fund, and the World Bank operationalize good governance and ACTA concepts within their own operations. The below describes the relevant institutions/mechanisms that these four IOs have in place.

### The World Health Organization (WHO)

The WHO is overseen by the Office of Internal Audit and Oversight (IAO) who inspects, monitors and evaluates the WHO’s internal control, financial management, and is also responsible for addressing any alleged breaches. It is composed of three divisions: (1) Internal Audit; (2) Inspection and Evaluation; (3) Investigation [36]. The IAO produces an annual report that is submitted to the World Health Assembly (WHA). The report includes summaries of investigations into allegations of misconduct at the WHO. In addition, the Panel of External Auditor also monitors the WHO’s operations in terms of its financial risk management and the efficacy of the organization’s internal control system. Its stated main objective is to *“further the coordination of the audits for which its members are responsible, and to exchange information on the audit methods and findings”* [36].

In the most recent report of the Internal Auditor to the WHA (2019), the overall ratings on operating effectiveness of internal controls from 2018 audits were reported as the same as 2017: a total of 81% of overall conclusions assessed as either “satisfactory” or “partially satisfactory with some improvement required” [37]. This overall rating is broken down further to reveal a significant increase of 78% in 2017 to 100% in 2018 in effectiveness of internal controls at regional offices, headquarters, and global cross-cutting areas [37]. However, the operating effectiveness of controls in country offices declined as equally significantly from 83% in 2017 to 60% in 2018 [37]. Given these findings, the IAO identified five priority areas: (i) improving assurance actions over Direct Financial Cooperation; (ii) strengthening direct implementation assurance activities, (iii) more robust vendor management and procurement documentation for goods and services; (iv) development of action plans to address human resource needs; and (v) improving resource mobilization for key underfunded programs [37].

WHO is also subject to an independent Expert Oversight Advisory Committee that is organized through its Executive Board. The purpose of the Committee is to advise the Program, Budget and Administration Committee and through it the Executive Board, in fulfilling their oversight advisory responsibility; on request, it also advises the Director-General on issues within its mandate. Last, a relatively recent addition (established in January 2014) to monitoring accountability within the WHO is the Office of Compliance, Risk Management and Ethics (CRE). CRE’s cited aims are to promote transparency and accountability through improving compliance, managing a risk framework, and promoting ethical values [37].

### The United Nations Development Programme (UNDP)

The Office of Audit and Investigations (OAI) is responsible for investigating alleged breaches [38], commensurate with the *UNDP Legal Framework for Addressing Non-Compliance with UN Standards of Conduct* and with the OAI Investigations Guidelines [39, 40]. The OAI also has an Investigator Hotline, which is “managed by an independent service provider on behalf of UNDP” and includes a confidential phone number and mailing address [41]. Any alleged staff misconduct within the UNDP is reported in the “Annual Reports of the Administrator on Disciplinary Measures and Other Actions Taken in Response to Fraud, Corruption and Other Wrongdoing” and are publicly available from 2011 [42–44]. Still, the reports are slim in terms of their details. This may be a result of OAI’s investigative guidelines, which states that investigations are strictly confidential [40]. Information regarding investigations are provided only to those with a “legitimate need to know,” such as affected staff members [40].

The OAI conducts regular internal audits of UNDP activity, which are publicly available on the UNDP website. Internal audits are undertaken by the OAI annually, reported to the Administrator and submitted to the Audit and Evaluation Advisory Committee for review [45]. Additionally, the OAI submits an annual report which highlights important observations from audits and investigations throughout the year which is presented to the UNDP Executive Board at an annual session in June [46]. In an effort to promote transparency, audit reports of member state offices and eligible donors since December 2012 have been publicly disclosed [47]. Prior to this, audits may be requested but must be kept confidential [48]. The UNDP also is overseen by an Ethics Office that is active in an investigation process conducted by OAI when staff members report that retaliatory actions have been taken against them for reporting a breach or providing information for an audit or investigation [49, 50]. The Ethics Office is an independent institution that reports directly to the Administrator of the UNDP [51].

The Audit and Evaluation Advisory Committee (AEAC) is tasked with working with the Administrator on oversight, financial management, reporting accountability, evaluation and internal and external audits [52]. The AEAC is an independent body and is composed of members, external to the UNDP. The mandate of this committee is highly ambitious: to *promote proper governance, high ethical standards, and the adoption of best practices*. The committee reviews annual reports and financial statements and provides recommendations to senior UNDP management [53]. The UN Board of Auditors prepares an audit report. Every year, it presents its report of the financial statements of the UNDP at the General Assembly through the Administrative and Budgetary committee and to the UNDP Executive Board [54].

The UNDP has a Transparency Portal (open.undp.org) in place, allowing public access to over 10,000 UNDP projects. The Transparency Portal publishes project data from 2012. In October 2019, the Portal had data about 4000 UNDP projects in 149 countries. Each project profile consists of information regarding its funding donor, outputs and, relevant documents (such as quarterly reports and project management documents) and purchase orders [55, 56].

The UNDP also publishes an Annual Report of the *Administrator on Disciplinary Measures and Other Actions taken in Response to Fraud, Corruption and Other Wrongdoings*. These reports are accessible from 2011 and summarize cases of misconduct and other violations of standards of conduct by UNDP staff, other personnel such as UN volunteers and contractors such as Services Contractors, and vendors [57]. Corruption cases are reported in the UNDP Financial Report and Audited Financial Statements, which are annual reports produced by the UN Board of Auditors (BoA). These reports contain cases of fraud and presumptive fraud disclosed by management and contains slightly more detailed descriptions of investigations of UNDP activities [58]. The cases outlined in the BoA report contains information of the region of the incident, time it was reported, a description of the incident, any remedial action against the persons involved, loss to the UNDP, and management action to deter recurrence of the incident. Reports are published on the UN Board of Auditor’s website dating back to 2000, however cases of fraud were only included in the report from 2008 onwards [59, 60]. Interestingly, the 2018 report diverts from previous structures of reporting, as it does not contain specific information on cases of fraud and corruption [61].

In 2018, a total of 520 cases concerning fraud, corruption and misconducted were reported, with 226 of these cases being carried over from 2017. This represents a slight increase in caseload, as 2014 identified 478. In 2018, 54% of these cases concerned financial irregularities, such as procurement fraud, theft and embezzlement. This number has remained relatively consistent throughout the past 5 years, with the exception of 2015 which saw an increase in financially related fraud. In 2018, cases primarily involved staff (28 substantiated cases) followed by vendors (25 substantiated cases) and service contractors (18 substantiated cases) [43].

### The Global Fund to Fight AIDS, Tuberculosis and Malaria (the Global Fund)

The Global Fund, like the UNDP, promotes the aspirational policy of zero tolerance for corruption and fraud. What is noteworthy is how many institutional checks the Global Fund has in place to reduce corruption risks and advance accountability and transparency. This makes sense given the intense scrutiny the Global Fund has come under given public exposure of corruption scandals in its operations.

The Office of the Inspector General (OIG) is responsible for conducting investigations and audits of Global Fund supported programs; all OIG reports are publicly accessible online, and the OIG even helps to disseminate information creating tweets about audits and investigations. Additionally, the OIG leads the **I Speak Out Now!** campaign, designed to encourage staff and grant implementers to report any suspected fraud and corruption incidents.

The Board of the Global Fund includes an “Audit and Finance Committee” and the “Ethics and Governance Committee” [62]. The Audit and Finance Committee oversee the Global Fund’s financial resources and audit and investigation reports that it receives from the Office of the Inspector General (OIG) while the Ethics and Governance Committee are responsible for “ethics-related matters” [63].

The OIG is responsible for overseeing Global Fund’s activities and investigations of alleged “Prohibited Practices [64].” The Global Fund defines its list of “Prohibited Practices” in its “Policy to Combat Fraud and Corruption [64].” Pursuant to the Global Fund’s whistleblowing policy, (updated in 2019), the OIG is also responsible for ensuring that there are adequate mechanisms for whistle-blowers to use [64]. The OIG is independent from the Secretariat and reports directly to the Board [65]. Importantly, the OIG also performs annual self-assessments and has external assessments every 3 years [66].

In 2018, the OIG planned and completed 19 audits, and screened 208 allegations, of which 64 allegations required more detailed assessment or investigation, resulting in the OIG opening 50 new investigations [66]. The majority of allegations came from whistle-blowers (107 allegations), followed by the Secretariat (35 allegations) and Fund Recipients (31 allegations) [66]. Notably, the OIG also performs proactive investigations that are “intelligence-led and does not rely on allegations from third parties or whistle-blowers” to detect wrongdoing before they escalate [67]. For example, in its most recently published proactive investigation, the OIG discovered non-compliance in the procurement of HIV rapid diagnostic test kits [67]. The OIG publishes detailed audits and investigations, which are available on the OIG’s website [68].

In 2014, the Global Fund also developed its “Ethics and Integrity Framework” and in May 2016, it established an Ethics Office and recruited an Ethics Officer [64]. The Ethics Officer reports to the Executive Director and the Board’s “Ethics and Governance Committee [64].” In 2018, the Ethics Office processed 245 cases, of which 177 cases were related to conflicts of interest [63]. Of the 177 conflict of interest cases, 82 were cleared, 73 had “mitigating measures” put in place, and 17 were not cleared [63]. Individuals or institutions who were not cleared either couldn’t take the position/assignment or had to step down [63]. Within the Global Fund, there has been significant improvement in reporting conflicts of interest. For example, 100% of Board members completed “Declarations of Interest” in 2019 compared to 76% completion in 2014 [63].

The Fund also implements Agreed Management Actions (AMAs) which are “an agreed course of action, decided jointly between the Secretariat and the Office of the Inspector General, to remedy an identified root cause, targeting specific portfolios where progress is needed [69].” The AMAs are published in audit and investigation reports completed by the OIG. The Global Fund tracks the progress it makes towards completing the AMAs in its “Joint AMAs Progress Reports” [69]. In its publication of November 14–15, 2018, the Global Fund reported that it had 68 open AMAs and 22 overdue AMAs, which represented an all-time low, demonstrating “significant progress” [69].

In audits and investigations where funds have been lost or were non-compliant with the grant agreement, an AMA is often to recover lost funds. Each year, the Global Fund Secretariat prepares a “Recoveries Report” for the Board that lists amounts owed and amounts recovered [70]. In November 2014, the Global Fund’s Board approved a measure of last resort called the “2-for-1 allocation reduction” to recover lost funds from countries that are “unwilling to budge” [70]. This measure allowed the Global Fund to withhold grants amounting to double the amount owed by the country [70]. For example, in the case of Bangladesh, the Global Fund spent 2.5 years negotiating with the government to recover US$ 2.1 million in lost funds, but there were no results [70]. Mark Dybul, (the former Executive Director of the Global Fund) in the end approved a US$ 4.2 million reduction in Bangladesh’s allocation [65]. As of June 2018, the “2-for-1 allocation reduction” penalty has been used seven times to recover US$ 12.7 million [70].

The OIG publishes audits and investigations on its website dating back to September 2008 [68]. There are 146 internal and country audit reports and 56 investigational reports [68]. What is noticeable is that the Global Fund has seen a change in trends of fraud and corruption from largely procurement fraud in 2014–2015 to a diverse range of fraud and corruption [66]. The OIG’s 2018 Annual Board Report shows that 80% of investigations related to procurement fraud between 2014 and 2015, compared to only 20% of investigations in 2018 [66]. Of the ten published 2018 investigations, two related to procurement fraud, two to embezzlement, two to “training/travel fraud,” two to drug theft, one to “salary kickbacks,” and one to “data fraud” [66].

### The World Bank Group (World Bank)

The World Bank, like the UNDP and the Global Fund, has an aspirational zero-tolerance policy towards corruption in its financed projects [23]. The Integrity Vice Presidency (INT) leads efforts to address corruption. It is an independent unit reporting directly to the President of the World Bank Group and overseen by the Audit Committee of the Executive Board [71]. INT mitigates risks of fraud and corruption through its investigations, sanctions and compliance, and prevention [71]. INT investigates both internal and external cases related to the World Bank’s sanctionable practices [71]. The investigation results produced are submitted to the World Bank Group President, as well as sent in referral reports to relevant national authorities [71]. This information is also provided in redacted reports to the Board of Executive Directors and posted on INT’s website [71].

Investigated cases found to have engaged in fraudulent, corrupt, collusive, coercive, or obstructive practices are sanctioned through a two-tiered system that includes the Office of Suspension and Debarment and the World Bank Group Sanction Board [71, 72]. In one recent example of this process, a sanction was imposed on the Government of Bangladesh as a result of the finding that there were collusive practices during the procurement of radiotherapy equipment for their Health Sector Development Program [73]. This sanction involved a posted letter of reprimand on the World Bank’s website for a six-month period starting on the date of the decision [73].

The World Bank states that sanctions like these are able to, “… *hold wrongdoers accountable for their misconduct and help deter others from engaging in similar behaviour*” [71]. Information about the Sanction System are detailed further in Annual Reports published on the World Bank’s website dating back to 2004 [71]. Their most recent report (2019), reports a total of 49 cases opened in 2019, compared to a total of 68 cases opened in 2018, and 51 cases opened in 2017 [74]. Of the total cases opened in 2019, 71% were related to fraud, 35% to corruption, 33% to collusion, 2% to coercion, and 6% to obstruction [74].

INT further mitigates risks of fraud and corruption through their Preventive Services Unit (PSU). The PSU was instituted in 2008 in response to requests from operational staff and a recommendation from the Independent Panel Review of INT [71]. The PSU prevents risks of fraud and corruption through applying the knowledge gained from investigative activities to the development and implementation of advice and training on the prevention of corruption in World Bank-financed projects [71].

The World Bank also supports its 189 member countries in its efforts to “build capable, efficient, open, inclusive, and accountable institutions” through its Global Governance Practices (GGP) [75]. The GGP recognizes the heightened pressures that the current COVID-19 pandemic is putting on governments as they attempt to respond to rapidly changing service delivery needs [75]. In order to build strong institutions, the GGP states broadly that it is working with its partners to aid them in addressing complex governance challenges, and working internationally to create global standards [75]. At present, the GGP reports briefly on the success of six such capacity building programs on its website [75]. For example, the World Bank supported Columbia in its reforms of its judicial branch. This included developing digital services, such as a digital transformation plan and a data governance strategy [75]. Another example is India’s Madhya Pradesh Citizens’ Access to Responsive Project which intends to increase public service access for women and other under-represented communities [75]. This involved the expansion of a number of “citizen service kiosks” in remote areas in order to improve coverage and provision of public services [75].

## Discussion and conclusions

The number and scope of ACTA efforts by IOs has had a marked increase over the last two decades [76]. While this paper provides an initial overview of such efforts employed by the UNDP, the Global Fund, the WHO, and the World Bank, it is by no means exhaustive. The searches conducted both within the academic literature and of the organizational websites were limited to material that was reported in or translated into English. Furthermore, this review did not examine all audit material provided by the IOs. Rather, it explored recent reports and extracted relevant and illustrative examples of the vast efforts IOs have made in recent years to demonstrate their accountability and transparency.

Our findings demonstrate that this is being done primarily through the adoption of accountability policies, frameworks, and public postings of their financial information and other relevant information on their websites, along with oversight of their operations by independent bodies. The above examples of the ACTA institutions/mechanisms the WHO, the Global Fund, the UNDP, and the World Bank have in place tend to be predominately audits and information sharing. Some mechanisms are clearly anti-corruption specific, while others provide broader mechanisms of accountability and transparency. This at the surface seems positive.

*Less apparent,* however, is how effective these various mechanisms are towards achieving institutional goals of accountability and transparency and anti-corruption, specifically in times of crisis such as the current COVID-19 pandemic. We need to know more about what mechanisms, whether they be preventative or retroactive, are being implemented when the usual ACTA procedures and protocols are bypassed to allow for more timely responses. Will the number of cases of corruption addressed by IOs increase over the course of the COVID-19 pandemic? If not, is this an indication that the retroactive ACTA mechanisms are ineffective? This is a difficult question to answer as there is a general absence of material demonstrating the efficacy of ACTA mechanisms. For example, if there are less reports of breaches and/or corruption, does this imply that the institutions/mechanisms are working satisfactorily? Or, is it the opposite case that these mechanisms are failing to catch them? Furthermore, if the institutions/mechanisms do catch them, do they have sufficient power and resources to effectively address the issue? The precise impacts of ACTA measures are challenging to discern and an important area for future research [25].

First, as noted earlier, there are no common definitions applied by IOs for accountability and transparency. Accordingly, it is challenging at best to make cross-comparisons amongst IOs. And, therein lies the thorny question: how do we measure their outcomes? What has complicated this enterprise further is as Rose-Ackerman underscores, good governance has become intertwined with the anti-corruption agenda and may in fact simply be “a convenient euphemism for corruption” [77]. This mixing of good governance with ACTA obfuscates the intent of these initiatives. Are they seeking to promote accountability and transparency or are they aiming to reduce the risk of corruption? Rose-Ackerman & Palifka importantly also ask the question “who is guarding the guard dogs?” [77].

Lastly, we are faced with challenges in terms of providing cross-institutional comparisons in this area as there is no common standard of transparency or accountability against which IOs can be evaluated [78]. Evaluations in IOs are missing techniques and methodologies and are “fragmented, non-comprehensive and non-integrated” [79, 80]. Of equal importance, international evaluations offices are often internal and missing critical independent reviews [80–83]. In short, there has clearly been a growth of efforts by IOs to demonstrate that they are accountable, transparent and taking steps to address corruption. All the same, it is timely and possibly urgent for IOs to step back and examine how they define concepts, such as good governance and ACTA, determine what they are doing in terms of advancing goals related to these areas, and most importantly, evaluate if their institutional “watch dogs” are achieving their intended purposes [25].

## Data Availability

Not applicable.

## Abbreviations

ACTA: Anti-corruption, accountability and transparency
IO: International organization
WHO: World Health Organization
UNDP: United Nations Development Programme
Global Fund: The Global Fund to Fight AIDS, Tuberculosis and Malaria
World Bank: The World Bank Group
WHA: World Health Assembly
